# Formation of the North Atlantic Warming Hole by reducing anthropogenic sulphate aerosols

**DOI:** 10.1038/s41598-022-27315-3

**Published:** 2023-01-02

**Authors:** Yuki Kusakabe, Toshihiko Takemura

**Affiliations:** 1 Interdisciplinary Graduate School of Engineering Sciences, Kyushu University, Fukuoka, Japan; 2 Research Institute for Applied Mechanics, Kyushu University, Fukuoka, Japan; 3 Present Address: Fujitsu Limited, Tokyo, Japan

**Keywords:** Atmospheric science, Climate change, Ocean sciences

## Abstract

The North Atlantic Warming Hole (NAWH) has been observed and predicted due to the increase in carbon dioxide (CO_2_) concentration. If sulphate aerosols, which have a cooling effect on the atmosphere, are reduced by air pollution control, the NAWH may form as it would if CO_2_ concentrations increased. In this study, sensitivity experiments using a coupled atmosphere–ocean-aerosol model were conducted by varying the amount of sulphur dioxide (SO_2_) emissions, a precursor of sulphate which is the primary anthropogenic aerosol in the atmosphere, to analyse the changes in the ocean temperature, salinity, and density. The results showed that although the spatial patterns of the NAWH due to the changes in SO_2_ emissions was similar to that due to the changes in the CO_2_ concentrations, the magnitude of the shifts in the ocean parameters due to the changes in SO_2_ emissions is larger even when changes in global mean temperature are comparable. This can be due to the spatial concentration of sulphate aerosols in the mid-latitudes of the Northern Hemisphere, resulting larger changes in the heat transport from the south on the Gulf Stream and the North Atlantic Current along with changes in freshwater inflow from the Arctic through the Labrador Sea.

## Introduction

The global mean surface temperature has increased by approximately 1.09 ℃ in 2011–2020 relative to 1850–1900 mean^1^. However, major databases of the surface temperature show that there is a region in the North Atlantic Ocean where the surface temperature increase is either extremely small or decreasing, called the North Atlantic Warming Hole (NAWH). The NAWH is also simulated in coupled atmosphere–ocean general circulation models (CGCMs)^1,2^. A simulation with an abrupt greenhouse gas increase by a CGCM shows the NAWH as a significant warming deficit within the North Atlantic Subpolar Gyre (NASG)^3^. The NAWH formation mechanism was investigated from large ensemble experiments with an earth system model^4^. According to the study, the NAWH is driven by an increased inflow of cold and low-salinity seawater from the polar regions causes stratification of the Labrador Sea, which weakens vertical mixing and reduces the heat supply from the subsurface layer, further reducing the heat content of the surface layer. As a result, cold surface water flows into the interior of the NASG, and weakens the Northern Recirculation Gyre (NRG), which is associated with the formation of the deep water in the Labrador Sea, changing the flow path of the Gulf Stream and the North Atlantic Current.

While changes in surface currents are important for the formation of the NAWH, the reduction in surface heat transport from low to high latitudes due to the weakening of the Atlantic Meridional Overturning Circulation (AMOC) is also thought to be related to the formation of the NAWH. Simulations with CGCMs share a common trend in which the AMOC weakens under warming scenarios^2,5^. However, the formation of NAWH precedes the weakening of the AMOC, and the physical relationship between them remains largely unknown^6^. A recent study^7^ showed that, in addition to the reduction in surface heat transport from the low latitudes, increased ocean heat transport from the NAWH into higher latitudes and a shortwave cloud feedback dominate the formation of the NAWH. Another recent study^8^ showed that the atmosphere can contribute approximately 50% of the observed cooling trend over the NAWH due to stronger local westerlies in response to external forcing that enhance heat loss from the ocean through turbulent heat fluxes. A past study^9^ also indicated that cold blobs, equivalent to the NAWH, are due to reduced heat transport by surface turbulent heat fluxes and ocean currents, and that AMOC slowdowns make only a marginal contribution to cold blobs. These suggested various theories indicate that the mechanism of the NAWH formation by the warming climate is still under discussion.

Sulphate aerosols are typical anthropogenic particles in the atmosphere and are mainly produced from sulphur dioxide (SO_2_) emitted by the combustion of fossil fuels. They have a global mean effective radiative forcing of − 0.90 ± 0.66 W m^–2^ estimated in the latest IPCC Assessment Report^1^, which means they have cooling effects on the Earth. Because recent air quality measures have led to a decrease in anthropogenic sulphate aerosols, it is expected that global warming will be accelerated. Although a few studies have shown that an increase in anthropogenic aerosols enhances the AMOC strength^5^, there have been no studies on analysing the NAWH formation due to changes in aerosol concentrations in detail. The distribution of anthropogenic sulphate aerosols is spatially heterogeneous, with high concentrations in the Northern Hemisphere, including Asia, Europe, and North America, and less in the Southern Hemisphere, which is much different from the CO_2_ distribution. In this study, the effect of changing sulphate aerosol concentrations on the formation of NAWH is investigated with a coupled atmosphere–ocean general circulation model, MIROC-SPRINTARS that has been developed by our research group. Black carbon (BC), which is a radiative-absorption aerosol, is also one of the major anthropogenic aerosols, but since most of the climate change due to BC is caused by atmospheric rapid adjustments^10^, this study, which primarily analyses oceanic changes, will focus on sulphate aerosols.

## Results

**Figure 1:**
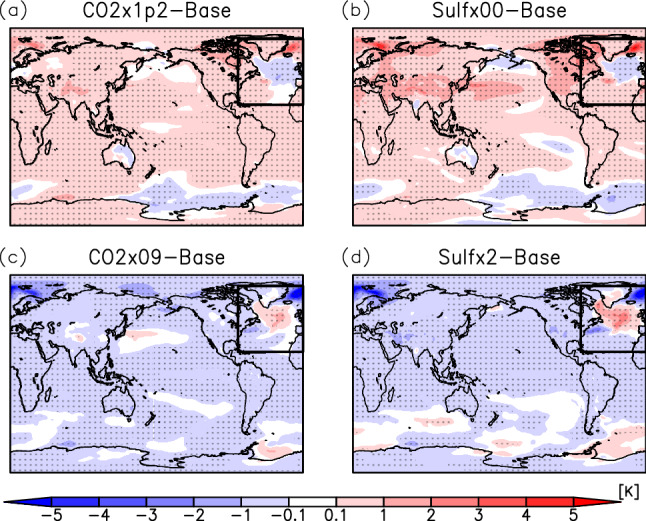
Anomalies of the annual mean surface air temperature for CO2 × 1p2 (**a**), Sulf × 00 (**b**), CO2 × 09 (**c**), and Sulf × 2 (**d**) experiments from the base experiment. Areas of dots indicate that the change is statistically significant. The area enclosed by the square represents the North Atlantic region defined in this study. The global mean temperature changes are + 0.32 K (**a**), + 0.41 K (**b**), − 0.28 K (**c**), and − 0.34 K (**d**), respectively. The maps were generated with GrADS 2.2.1 (URL: http://cola.gmu.edu/grads/).

In this study, sensitivity experiments were conducted with no anthropogenic SO_2_ emissions (Sulf × 00) and double anthropogenic SO_2_ emissions (Sulf × 2) compared to those in the base experiment; and for comparison, additional experiments were also carried out wherein CO_2_ concentrations of 0.9 (CO2 × 09) and 1.2 (CO2 × 1p2) times, respectively, were changed to produce nearly equivalent global mean surface temperature changes (see “Methods” in detail). When global warming occurs, the NAWH appears in the North Atlantic region (Fig. 1a,b), while the reversed NAWH occurs in the same region under global cooling (Fig. 1c,d). The distribution of the sea surface temperature (SST) anomalies is similar to that of surface air temperature anomalies, and the NAWH appears in the same region, as shown later. Compared to the CO_2_ sensitivity experiments, the SO_2_ emission change results in larger temperature changes in the North Pacific, Asia, Europe, and the United States, which are regions with high industrial activity.

**Figure 2:**
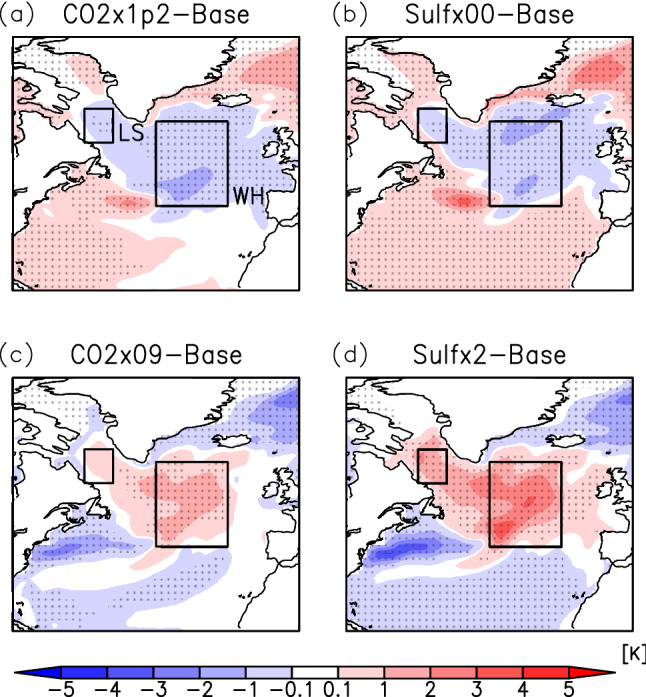
Anomalies of the annual mean sea surface temperature for CO2 × 1p2 (**a**), Sulf × 00 (**b**), CO2 × 09 (**c**), and Sulf × 2 (**d**) experiments from the base experiment in the North Atlantic. Areas of dots indicate that the change is statistically significant. LS and WH show the Labrador Sea and NAWH regions, respectively. The maps were generated with GrADS 2.2.1 (URL: http://cola.gmu.edu/grads/).

**Figure 3:**
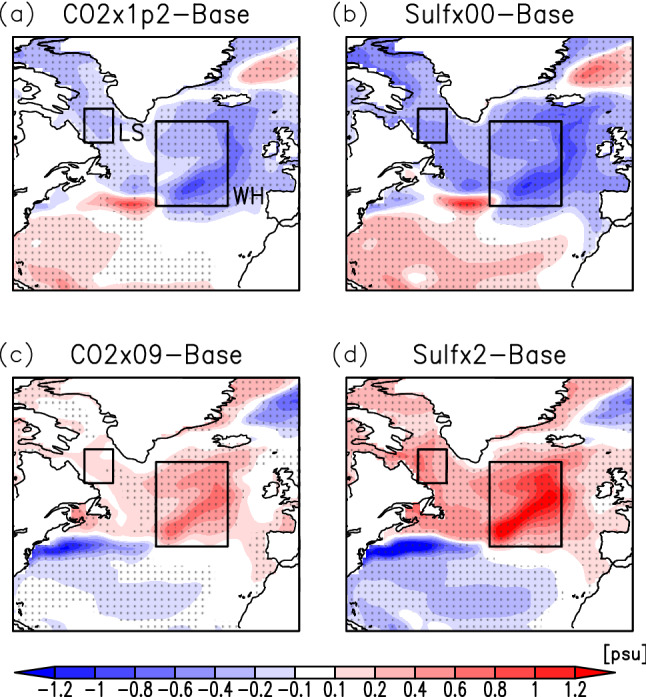
Same as Fig. 2 but for the annual mean sea surface salinity. The maps were generated with GrADS 2.2.1 (URL: http://cola.gmu.edu/grads/).

Figures 2 and 3 show the anomalies in SST and sea surface salinity (SSS), respectively, in the North Atlantic region (0°–80°W, 20°–80°N). In the NAWH region, the temperature anomaly is reversed from the trend of global change in all the experiments. Under global warming (Sulf × 00), SST cooling in the Labrador Sea also occurs in addition to the formation of the NAWH inside the NASG (Fig. 2b). The distributions of the regions where temperature changes are reversed are almost the same for CO2 × 1p2 (Fig. 2a) as Sulf × 00. The reversed NAWH with global cooling (Sulf × 2) also has a geographic distribution similar to that of the NAWH (Fig. 2d). The anomaly in the SSS from the base experiment decreases north of 40°N with global warming and increases with global cooling (Fig. 3b,d). This is due to changes in the amount of high-salinity surface seawater transported from the south by the North Atlantic Current. However, as discussed later, since the current velocity from the Labrador Sea to the interior of the NASG is enhanced with global warming, the decrease in SSS during the formation of the NAWH can also be due to an increase in the inflow of low-salinity seawater from the polar region.

The SST anomalies of the global and regional mean in the NAWH region are shown in Table 1. The global mean SST anomalies show the similar level of warming and cooling between CO2 × 1p2 and Sulf × 00 and between CO2 × 09 and Sulf × 2, respectively. However, the temperature increase inside the NAWH region with global cooling is approximately twice as large for Sulf × 2 than for CO2 × 09. The reason for this difference can be explained by the fact that the sulphate aerosols produced from SO_2_ are more unevenly concentrated in the mid-latitudes of the Northern Hemisphere than CO_2_. The NAWH region is consistent with areas of air pollutant outflow from North America. This will be discussed in more detail later, along with a discussion of the main factors in the formation mechanism of NAWH.

**Table 1:** Anomalies of the annual mean sea surface temperature averaged over global and the NAWH region for each sensitivity experiments from the base experiment.

	∆Sea surface temperature (K)			
	CO2 × 1p2	Sulf × 00	CO2 × 09	Sulf × 2
Global	+ 0.22	+ 0.23	− 0.19	− 0.21
NAWH	− 0.75	− 0.62	+ 0.83	+ 1.68

**Figure 4:**
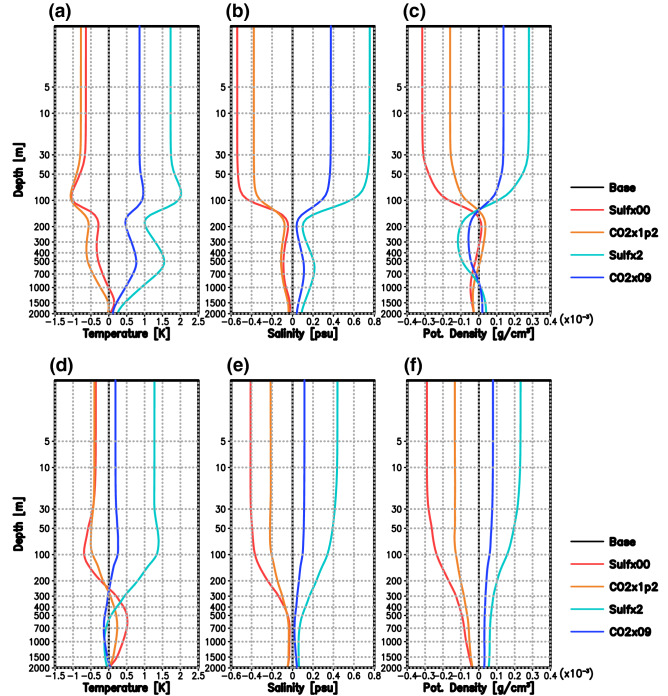
Anomalies of annual mean vertical profiles of (**a,d**) potential ocean temperature (K), (**b,e**) salinity (psu), and (**c,f**) potential density (g cm^–3^) averaged in the (**a–c**) NAWH region (20°–40°E, 40°–60°N) and (**d–f**) Labrador Sea (52°–60°E, 55°–63°N) for each experiment from the base experiment.

The vertical profiles of ocean temperature, salinity and density averaged in the NAWH region are shown in Fig. 4a–c. The NAWH and reversed NAWH are clearly shown to occur mainly in the surface layer shallower than 100 m depth (Fig. 4a). The increase in ocean temperature causes a decrease in density, and on the other hand, the increase in salinity causes an increase in density. Therefore, the effect of the salinity change is dominant in the NAWH region (Fig. 4b,c). The effect of this density change can alter the vertical mixing in the NAWH region and weaken the heat exchange with the warm seawater in the subsurface layer, leading to further cooling of the SST.

It should be noted here that the changes in temperature, salinity, and density in the NAWH region are larger under adjustments to sulphate aerosol concentration changes than under CO_2_ concentration changes. While there is little difference between them (Sulf × 00 and CO2 × 1p2) in regards to temperature change under global warming, the difference in temperature change under global cooling between Sulf × 2 and CO2 × 09 is clear. The change in salinity and the associated change in density are larger in the case of sulphate aerosol concentration changes in both the NAWH and reversed NAWH cases. This can be attributed to the fact that anthropogenic sulphate aerosols are concentrated in the mid-latitudes of the Northern Hemisphere. Temperature changes in the mid-latitudes of the Northern Hemisphere due to anthropogenic sulphate aerosols are then greater than in other latitudinal zones^11^.

### Changes in the North Atlantic heat flux

**Figure 5:**
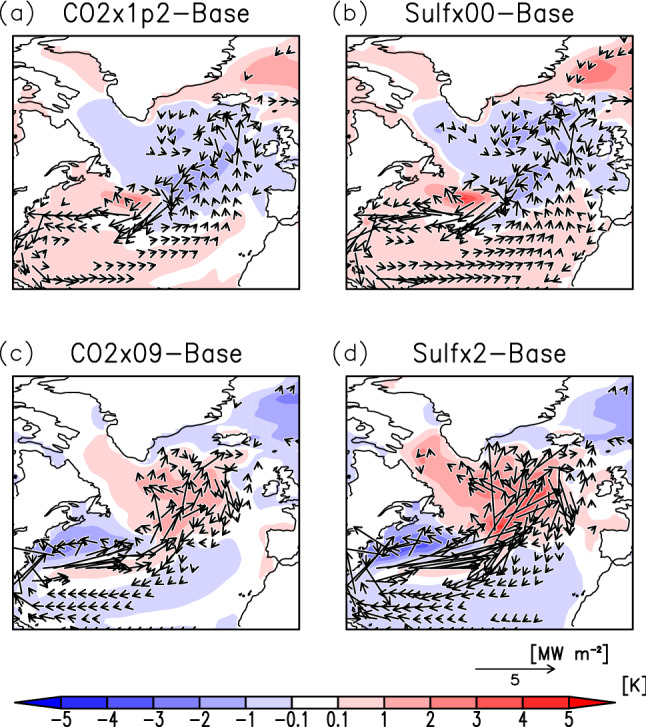
Anomalies of annual mean horizontal heat flux (vectors) with ocean temperature (colours) for CO2 × 1p2 (**a**), Sulf × 00 (**b**), CO2 × 09 (**c**), and Sulf × 2 (**d**) experiments from the base experiment in the North Atlantic. They are averaged below 100 m depth. Anomalies of the horizontal heat flux below 0.25 MW m^–2^ are not shown. The maps were generated with GrADS 2.2.1 (URL: http://cola.gmu.edu/grads/).

Figure 5 show the distribution of horizontal heat flux and ocean temperature anomalies relative to the base experiment averaged below 100 m depth. The formation of the NAWH with global warming can be attributed to the change in heat transport from the south to the interior of the NASG due to the change in the flow path of the North Atlantic Current and the increase in the inflow of cold water from the surface layer of the Labrador Sea into the interior of the NASG^4^. On the other hand, during the formation of the reversed NAWH under global cooling condition, caused by the decrease in CO_2_ concentration and increase in SO_2_ emissions, the flow path of the North Atlantic Current is strengthened towards the interior of the NASG, which increases the inflow of warm seawater from the south. Then, the surface water inside the reversed NAWH flows into the inner part of the Labrador Sea by the enhanced NASG, causing a positive temperature change in the Labrador Sea. Although heat fluctuates in the ocean occur vertically, their contribution to the formation of NAWH is negligible because the amount of change is more than three orders of magnitude smaller than that of horizontal heat fluxes. Additionally, the change in horizontal heat flux between the Labrador Sea and the NAWH region is less than 0.5 MW m^–2^, which is less than the change in heat flux due to changes in the Gulf Stream and North Atlantic Current, and therefore it has a limited role in NAWH formation (Fig. 5a,b). With global cooling, the enhanced NASG increases the heat flux from the reversed NAWH region to the Labrador Sea. The change in the horizontal heat flux is approximately twice as large in the Sulf × 2 experiment (Fig. 5d) than in the CO2 × 09 experiment (Fig. 5c), which is consistent with the larger temperature anomaly in Sulf × 2 than in CO2 × 09 (Table 1).

The AMOC weakens with global warming and strengthens with global cooling, resulting in changes in oceanic heat transport (Fig. S1). In particular, the AMOC stream function strengthens approximately twice in Sulf × 2 than in CO2 × 09 (Fig. S1c,d), which is consistent with the difference in the regionally averaged temperature change in the NAWH region under global cooling conditions (Table 1). This confirms that heat transport from the south in the surface layer is dominated by the AMOC and is consistent with changes in the Gulf Stream and the North Atlantic Current.

In the case of global cooling, reversed NAWH appears stronger in the increased SO_2_ emissions (Sulf × 2) than in the decreased CO_2_ concentration (CO2 × 09), while in the case of NAWH associated with global warming, the difference between increased CO_2_ concentration (CO2 × 1p2) and decreased SO_2_ emissions (Sulf × 00) is not clear (Table 1). The reason can be that although a difference in the strength of the AMOC stream function changes appears between CO2 × 1p2 and Sulfx00 (Fig. S1a,b), there is little difference between them for the change in horizontal heat flux in the surface layer, the main mechanism for NAWH formation (Fig. 5a,b). That is, the degree of weakening of the North Atlantic Current flux into the interior of the NASG due to the northward shift of the Gulf Stream caused by global warming is similar in both cases. Reduced SO_2_ emission means that spatial heterogeneity in the radiative forcing due to sulfate aerosols is eliminated, thus nonlinear changes are mitigated not only in the atmosphere but also in the ocean surface layer directly affected by the atmosphere.

The changes in horizontal heat fluxes are mainly due to changes in the velocity and the path of the Gulf Stream and the North Atlantic Current, and the changes in the currents are also due to changes in the Deep Western Boundary Current (DWBC), which is the Labrador Deep Water Current that flows southward around the Island of Newfoundland^6^. The DWBC weakens with global warming and strengthens with global cooling (Fig. S2). The DWBC has a significant influence on the path of the Gulf Stream through changes in the NRG vorticity.

Figure 4d–f show the vertical profiles of anomalies of temperature, salinity, and density from the base experiment averaged in the Labrador Sea. The effect of salinity change is the primary factor that changes the density of seawater, as seen in the NAWH region (Fig. 4a–c). The salinity changes at depths below 200 m are approximately twice as large with global warming and approximately three times as large with global cooling in the sulphate sensitivity experiments. This can be related to the fact that the changes in the mixed layer depth in the Labrador Sea are greater in the sensitivity experiments for sulphate concentration than for CO_2_.

### Changes in freshwater fluxes in the North Atlantic

Freshwater fluxes were analysed to investigate the causes of low salinization in the surface waters of the Labrador Sea. The freshwater and horizontal freshwater fluxes were defined as FW = 1–(S/S_ref_) and **FW** = FW⋅**V**, respectively, where S is the salinity of seawater, S_ref_ = 34.7 psu, and **V** is the current horizontal velocity vector^4^. The anomaly of the freshwater flux from the base experiment in each sensitivity experiment was analysed by decomposing it into two components, one originating from the change in freshwater volume and the other from the change in current velocity, as follows.

1 $$\Delta {\mathbf{FW}} = \, \Delta ({\text{FW}} \cdot {\mathbf{V}}) \, = \, \Delta {\text{FW}} \cdot {\mathbf{V}} + {\text{ FW}} \cdot \Delta {\mathbf{V}} .$$

Freshwater fluxes are averaged below 100 m depth in the analysis.

With global warming, the freshwater inflow from the Arctic increases mainly due to the flux through the Canadian Arctic Archipelago (CAA) into the Labrador Sea and the inner NASG (Fig. S3a). It is shown that the freshwater volume increases from the Arctic Ocean into Baffin Bay through the CAA (Fig. S3b) and that the increase in current velocity southward in Baffin Bay increases the freshwater flux into the Labrador Sea (Fig. S3c). Conversely, with global cooling, the freshwater flux from the Arctic Ocean and Labrador Sea into the NASG decreases (Fig. S3d–f).

**Figure 6:**
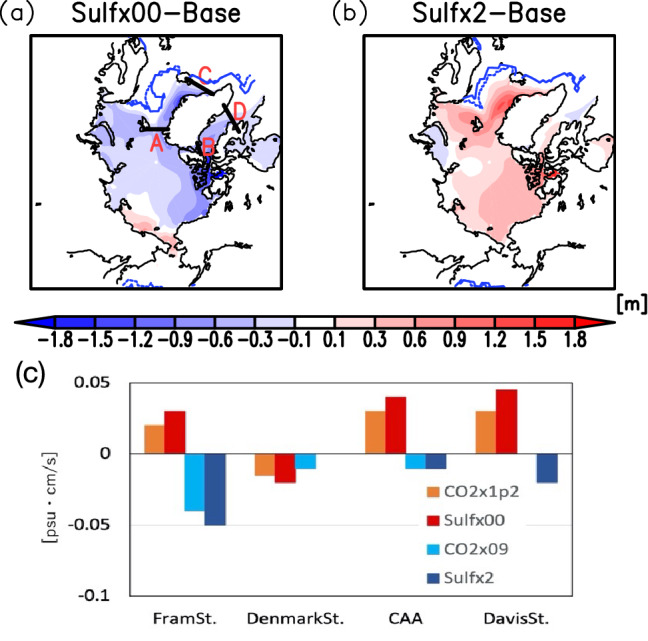
Anomalies of the annal mean Arctic sea ice thickness for Sulf × 00 (**a**) and Sulf × 2 (**b**) experiments from the base experiment. The dashed and solid blue lines are the locations of the annual mean sea ice thickness of 0 m in the base and each sensitivity experiments, respectively. The letters A, B, C, and D in (**a**) are the locations of the Fram Strait, Canadian Arctic Archipelago (CAA), Denmark Strait, and Davis Strait, respectively. The maps were generated with GrADS 2.2.1 (URL: http://cola.gmu.edu/grads/). The annual mean southward freshwater flux through each strait below 100 m depth is in (**c**).

Figure 6a and b show the change in sea ice thickness in the Arctic. With global warming, there are particularly large decreases north of Greenland and around the CAA. Conversely, with global cooling, large increases in sea ice thickness occur in the Greenland Sea and around the CAA. With global warming, the melting of arctic sea ice brings freshwater into the North Atlantic through two major entry points: the Fram Strait and the CAA. Figure 6c shows the meridional components of the freshwater fluxes through the surface layer of the four straits (Fram Strait, CAA, Denmark Strait, and Davis Strait shown in Fig. 6a). The southward freshwater flow through the Fram Strait and the CAA is enhanced by the melting of sea ice in the Arctic Ocean. The flux through the Fram Strait is divided into the flow to the North Atlantic through the Denmark Strait and northward again to the Barents Sea, of which the component flowing into the North Atlantic is reduced. The southward freshwater flux through the CAA is found to pass through the Davis Strait and into the Labrador Sea without changing its magnitude. With global cooling by increasing sulphate aerosols (Sulfx2), the change in freshwater flow through the Denmark Strait is also very limited.

Therefore, the freshwater supply from the Arctic region to the North Atlantic Ocean is dominated by the change in the freshwater flux through the CAA. The freshwater fluxes in the North Atlantic region from the melting of sea ice are more than one order of magnitude larger than other freshwater flux changes, including precipitation, evaporation from the sea surface, and river inflow.

## Discussion and conclusions

In this study, the formation of the NAWH and reversed NAWH due to changes in sulphate aerosol concentration, i.e. changes in SO_2_ emissions, was analysed from simulated results with the atmosphere–ocean-aerosol coupled general circulation model MIROC-SPRINTARS. Although the spatial NAWH and reversed NAWH patterns due to the change in sulfate aerosol concentrations were similar to the change in CO_2_ concentrations, there were some differences in the changes in temperature, salinity, and density under similar global mean surface air temperature changes. With global cooling, the temperature increase in the NAWH region was approximately twice as large in the Sulf × 2 experiment than in the CO2 × 09 experiment, which corresponds to the anomaly of the horizontal heat flux being approximately twice as large in the Sulf × 2 experiment. This can be due to the spatial heterogeneity of sulphate aerosol concentrations that are abundant in the mid-latitudes of the Northern Hemisphere, where industrial activity is prevalent.

The simulations indicated that changes in the pathways and velocities of the Gulf Stream and North Atlantic Current are important for the formation of the NAWH and reversed NAWH with changes in sulphate aerosol concentrations. With global warming, the northward shift of the Gulf Stream reduces the horizontal heat flux into the NASG, resulting in the formation of the NAWH. It was confirmed that the change in the DWBC is important for the change in the Gulf Stream and that the shift in seawater density in the Labrador Sea is caused by the change in salinity of seawater. The simulation showed that the density change is larger in the SO_2_ emission change than in the CO_2_ concentration change. The lowering salinity of the surface layer in the Labrador Sea with global warming is mainly due to the increase in melting arctic sea ice, resulting in the increase in southward freshwater flux through the CAA and the Davis Strait.

The impacts of the NAWH on the atmosphere are discussed here. The spatial distribution of the anomaly of evaporation from the sea surface (Fig. S4a,d) and precipitation (Fig. S4b,e) in the North Atlantic also corresponds well with the SST anomaly (Fig. 3a,c). Approximately 50% of the anomaly in the air-sea heat flux is accounted for by the anomaly in the latent heat due to evaporation from the sea surface. The change in the ice water path (Fig. S4c,f) is also in agreement with the distribution of temperature, latent heat from the sea surface, and precipitation anomalies due to the NAWH and reversed NAWH. The fact that the change in the ice water path in the Sulf × 2 experiment is approximately twice as large as that in CO2 × 09 (not shown) is consistent with the reversed NAWH being approximately twice as strong (Table 1). However, the change in the liquid water path over the North Atlantic seems to be dominated by the inflow of moist air from subtropical regions with global warming. The climate change mechanisms due to aerosols through aerosol-radiation and aerosol-cloud interactions are more complex than those due to CO_2_. The next step of research should be a detailed analysis of which processes of atmospheric perturbation associated with changes in aerosol concentrations contribute to the formation of NAWH.

## Methods

The model used in this study is the coupled atmosphere–ocean-aerosol general circulation model MIROC-SPRINTARS. MIROC6^12,13^, the latest version of MIROC, is used with a horizontal resolution of T85 (approximately 1.4° × 1.4° in longitude and latitude) and 40 vertical layers in the atmosphere, a horizontal resolution of 1 degree in longitude and 0.5–1 degree in latitude direction, and 40 vertical layers in the ocean. The aerosol module SPRINTARS^14–16^, which is coupled to MIROC, calculates not only the transport processes of aerosol species and their precursors, such as sulphate, black carbon, organics, sea salt, and soil dust but also their aerosol-radiation and aerosol-cloud interactions. The transport processes to be calculated are emission, advection, diffusion, sulphur chemistry, wet deposition, and dry deposition.

To investigate how surface temperature changes in the North Atlantic Ocean with increasing or decreasing fuel-derived sulphate aerosols, equilibrium sensitivity experiments were conducted by varying the emission of SO_2_, a precursor of sulphate aerosols, by a factor of 0 (Sulf × 00) and 2 (Sulf × 2) relative to the present emissions based on the EDGAR-HTAP database in 2014^17^. Equilibrium sensitivity experiments were also conducted with the present SO_2_ emissions by varying the CO_2_ concentration by a factor of 1.2 (CO2 × 1p2) and 0.9 (CO2 × 09) from the present concentration (368.86 ppm in 2000), in which the global mean surface temperature change would be comparable to Sulf × 00 and Sulf × 2, respectively. These simulated results were analysed with anomalies from the base experiment using the present SO_2_ emissions and CO_2_ concentration. The other experimental settings are the same as in earlier studies^11,18^, which are derivative experiments from the Precipitation Driver and Response Model Intercomparison Project (PDRMIP)^19^. In equilibrium experiments using the coupled atmosphere–ocean model, it is necessary to analyse the data after the climate has reached equilibrium for several decades. In this study, time-averaged data were analysed for the latter half of 50 years out of 100-year integration.

## Supplementary Information

Supplementary Figures.

## Data Availability

The datasets used and/or analysed during the current study available from the corresponding author on reasonable request.
