# Supplementary material for: Formation of the North Atlantic Warming Hole by reducing anthropogenic sulphate aerosols
Source: Sci Rep. 2023 Jan 2;13:8. doi: 10.1038/s41598-022-27315-3 (PMC9807570; doi:10.1038/s41598-022-27315-3)
Supplement: Supplementary file 1 — Supplementary Figures. [file 41598_2022_27315_MOESM1_ESM.pdf]

Supplementary Information

Formation of the North Atlantic Warming Hole by reducing anthropogenic sulphate aerosols

Yuki Kusakabe[1] and Toshihiko Takemura[2]*

[1]Research Institute for Applied Mechanics, Kyushu University, Fukuoka, Japan

[2]Now: Meteorological Research Institute, Japan Meteorological Agency, Ibaraki, Japan

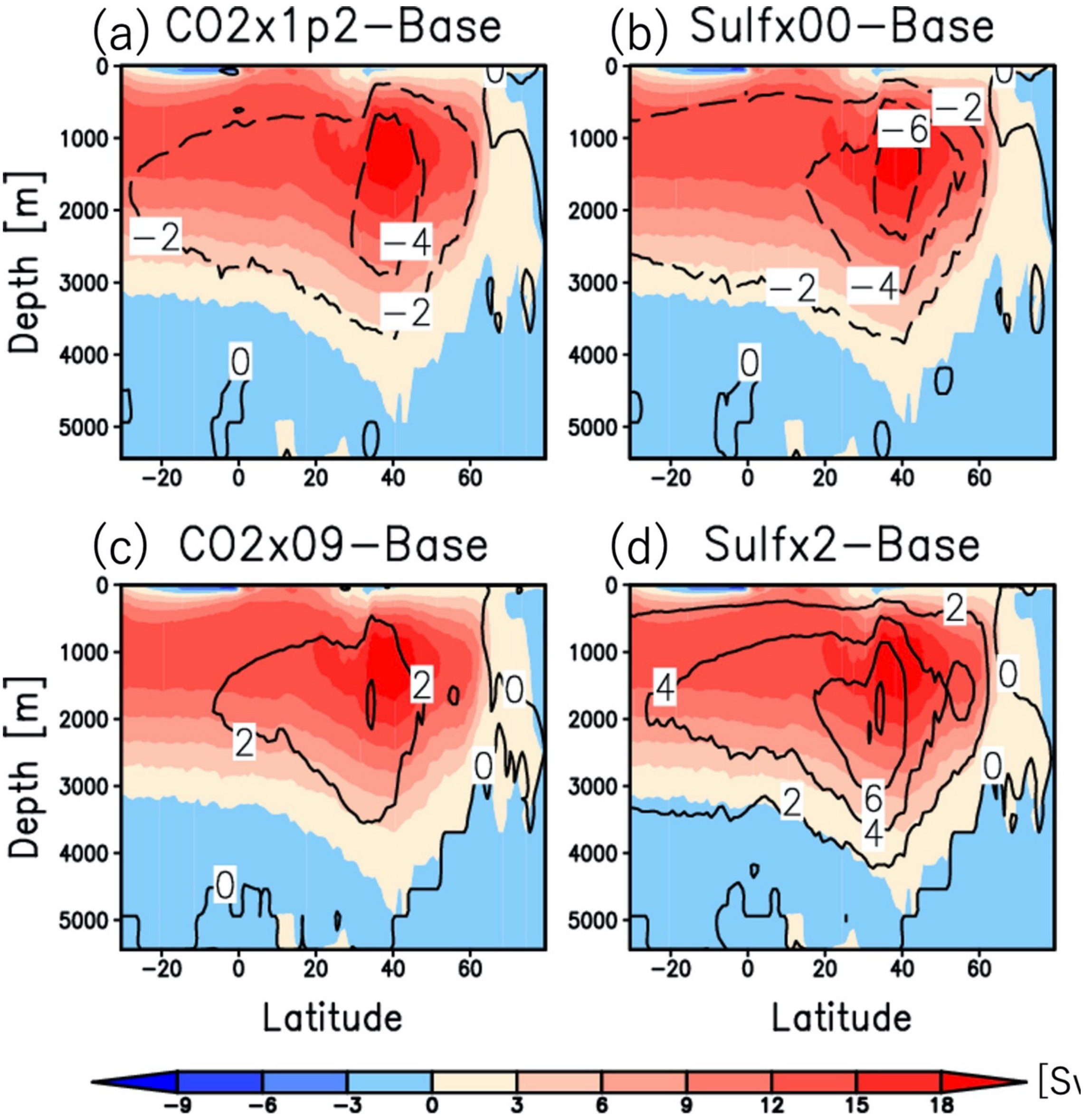

Fig. S1 | Anomalies of the AMOC stream function (black lines). (a) CO2x1p2, (b) Sulfx00, (c) CO2x09, and (d) Sulfx2 experiments. The colour shades indicate the AMOC stream function in the base experiment. The maps were generated with GrADS 2.2.1 (URL: http://cola.gmu.edu/grads/).

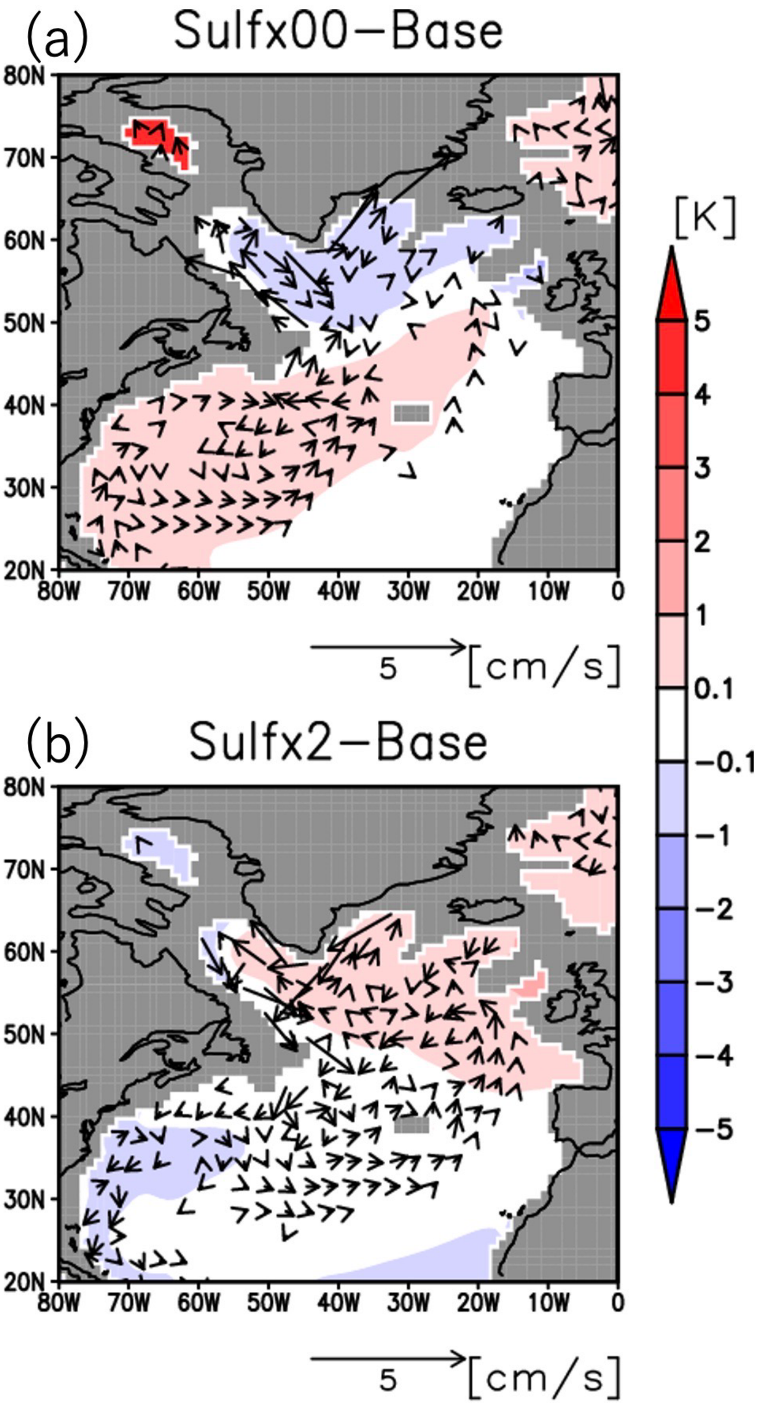

Fig. S2 | Anomalies of the ocean temperature (colours) and horizontal current velocity (vectors) at a depth of approximately 2000 m in the North Atlantic Ocean. (a) Sulfx00 and (b) Sulfx2 experiments. Vectors with magnitudes less than 0.1 cm s^{-1} are excluded. The maps were generated with GrADS 2.2.1 (URL: http://cola.gmu.edu/grads/).

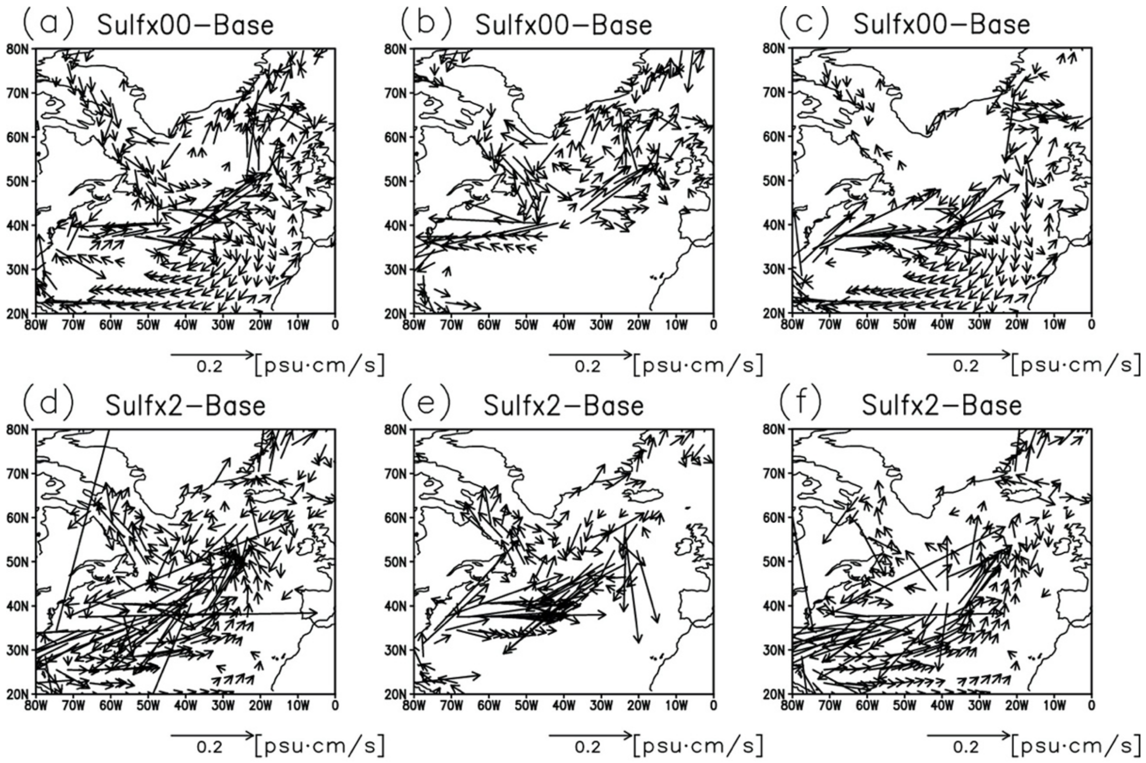

Fig. S3 | Anomalies of horizontal freshwater fluxes. The total horizontal freshwater fluxes are shown in (a) and (d), and their components of freshwater amount and flow velocity are shown in (b, e) and (c, f), respectively, in the Sulfx00 and Sulfx2 experiments. All vectors with magnitudes less than 0.02 psu cm s^{-1} are excluded. The maps were generated with GrADS 2.2.1 (URL: http://cola.gmu.edu/grads/).

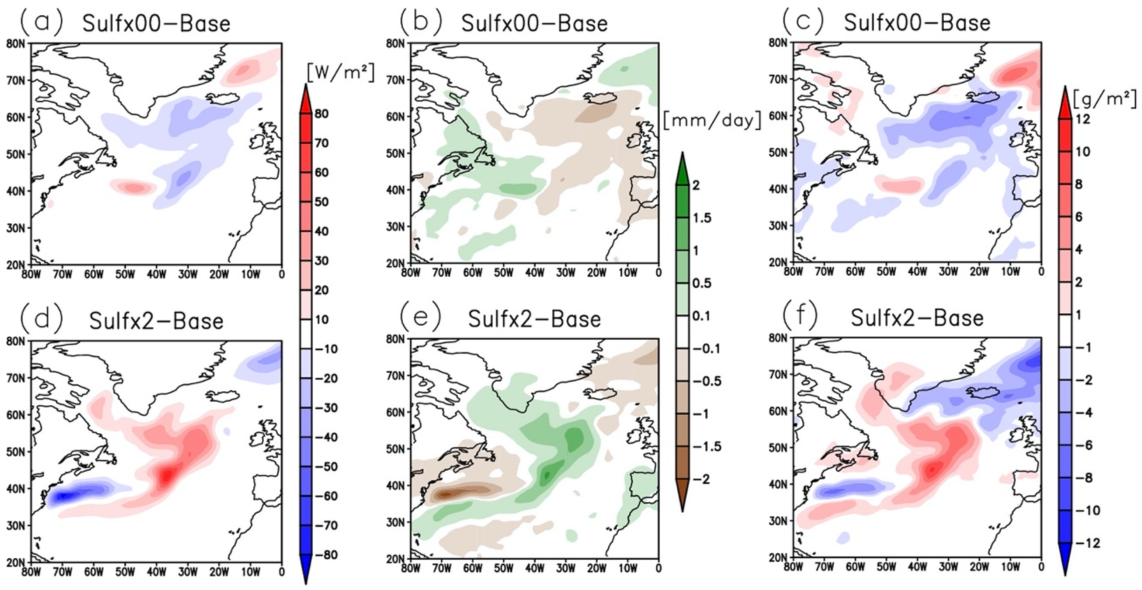

Fig. S4 | Anomalies of (a, d) latent heat from the sea surface, (b, e) precipitation, and (c, f) ice water path. (a–c) Sulfx00 and (d–f) Sulfx2 experiments. The maps were generated with GrADS 2.2.1 (URL: http://cola.gmu.edu/grads/).
